# Targeting Amino Acid Metabolic Vulnerabilities in Myeloid Malignancies

**DOI:** 10.3389/fonc.2021.674720

**Published:** 2021-05-20

**Authors:** Livingstone Fultang, Luciana Gneo, Carmela De Santo, Francis J. Mussai

**Affiliations:** Institute of Immunology and Immunotherapy, University of Birmingham, Birmingham, United Kingdom

**Keywords:** amino acids, metabolism, therapy, myeloid dysplasia, myeloid neoplasia

## Abstract

Tumor cells require a higher supply of nutrients for growth and proliferation than normal cells. It is well established that metabolic reprograming in cancers for increased nutrient supply exposes a host of targetable vulnerabilities. In this article we review the documented changes in expression patterns of amino acid metabolic enzymes and transporters in myeloid malignancies and the growing list of small molecules and therapeutic strategies used to disrupt amino acid metabolic circuits within the cell. Pharmacological inhibition of amino acid metabolism is effective in inducing cell death in leukemic stem cells and primary blasts, as well as in reducing tumor burden in *in vivo* murine models of human disease. Thus targeting amino acid metabolism provides a host of potential translational opportunities for exploitation to improve the outcomes for patients with myeloid malignancies.

## Introduction

Amino acid metabolism is fundamental to several biological processes affecting cell survival and growth. In all cell types, amino acids (AAs) are needed for protein, lipid, and nucleic acid synthesis. AAs also serve as a major source of nitrogen and carbon units in the biosynthesis of other low molecular weight compounds such as polyamines. In many cancers, AAs are alternative fuels for energy production and are critical for redox homeostasis and the production of secondary signaling and regulatory factors. To meet the added demand for AAs to support rapid autonomous cell growth, myeloid neoplasms and acute leukemias are metabolically rewired to shunt the energy dependent pathways for intracellular re-synthesis in favor of their import from extracellular sources. The resulting dependency on exogenous as well as endogenous AAs entails a significant targetable vulnerability currently being exploited in the development of new therapies.

Myeloid malignancies are a heterogeneous group of disorders arising from clonal expansion of hematopoietic stem cells (HSCs) or myeloid precursors often bearing multiple genetic mutations that disrupt normal myeloid cell maturation and differentiation (1). They include myelodysplastic syndromes (MDS), myeloproliferative neoplasm (MPN) disorders (such as chronic myelomonocytic leukemia (CML)) and acute myeloid leukemia (AML) with various genetic abnormalities. All these conditions are associated with uncontrolled proliferation of immature non-functional myeloid cells in the bone marrow that rapidly leads to bone marrow failure and extensive immune dysregulation. Critical to the clonal evolution of leukemia is the generation of a leukemic stem cell (LSC) line capable of linking the cellular expression patterns of proteins (enzymes, transporters) to nutrient availability for energy production and molecular building blocks such as amino acids. In this review we will delineate the specific vulnerabilities of amino acid metabolism described for myeloid malignancies and explore the growing list of therapeutics specifically designed to target such vulnerabilities (**Table 1**).

**Table 1:** Drugs targeting amino acid metabolism in myeloid malignancies.

Amino acid	Drug(s)	Target	Disease type or model	Response	Reference
Methionine	PF-9366	MAT2	AML bearing MLL-AF9 fusion protein	Reduction in cell viability	(9, 10)
	AEP, AMB, Cycloleucine	MAT1, MAT2, MAT3	Untested in myeloid Malignancies	N/A	(11–13)
	Pinometostat	SAM	AML blast from adult and pediatric patients	Reduction in H3K9me	(14)
	GSK-343	SAM	AML cell line Kasumi	Reduction in H3K27me3, G_0_/G_1_ cell cycle arrest	(15)
	LLY-283	SAM	AML cell lines MOLM-13 and MV411	Blocks cell proliferation *in vitro*	(16)
	PEG-rMETase	Extracellular methionine depletion	Untested in myeloid malignancies	N/A	(24)
Cysteine	AOAA	CBS	CML cell line K562	Inhibition of proliferation and induction of apoptosis *in vitro*	(21)
BCAAs	ERG240	BCAT1	Myeloid cells	Reduces oxygen consumption, glycolysis and itaconate levels	(30, 31)
	BCH	LAT1, LAT2, LAT3	AML cell lines HL60 and NB40; CML cell line K562	Reduces growth rate *in vitro*; downregulates S6RP phosphorylation.	(39, 40)
	JPH203, SKN103	LAT1	Untested in myeloid malignancies	N/A	(38, 41)
Tryptophan	Indoximod	IDO	Patient derived AML blast.	Reversed blast-mediated suppressive effect on T cell proliferation *in vitro*	(44)
			Newly diagnosed AML patients	Ongoing phase I/II trial (NCT02835729) in combination with Cytarabine	N/A
	Epacadostat	IDO	Patients with advanced MDS after Azacytidine treatment	Stable disease in 80% of cases. Marginal reduction in MDSCs and Tregs	(58)
	Navoximod	IDO	Co-culture experiments with human monocytes derived dendritic cells and T cells	Restores T-cell proliferation *in vitro*	(59)
	Lirondostat	IDO	Patients with MDS or AML	Ongoing Phase I/II trial (NCT02835634) in combination with Nivolumab	N/A
Glutamine	CB839	GLS	Patient derived AML blasts	Reduces intracellular glutamate titers with corresponding reduction in cell viability	(63–65)
			Patients with advanced MDS after Azacytidine treatment	70% complete response. Sensitivity is increased when administered in conjunction with the FLT3 inhibitor AC220	(66, 68)
	V9302	ASCT2	Blasts isolated from bone marrow of transgenic mouse model of MLL-AF9 induced AML	V9302 leads to reduction in glutamine and leucine uptake, inhibition of mTOR and induction of cell death.	(69)
Cysteine	Erastin	xCT	AML cell lines	Increased intracellular ROS production and cell death by ferroptosis	(72, 73)
Serine, Glycine	WQ2101	PHGHD	AML cell lines MV411, MOLM13, PL21	Induction of apoptosis only in cells harboring FLT3-ITD mutations	(77).
	PHGHD-Hit, BI4924, NCT502, NCT502	PHGHD	Untested in myeloid malignancies	N/A	(78, 79)
	Pemetrexed, Lometrexol, Methotrexate, Raltitrexed	SHMT2	Recombinant human SHMT2	Up to 60% reduction in SHMT2 activity	(83)
	Compound 12.2	SHMT2	CML Cell line HAP1	Reduction in cell viability	(82)
	AGF291, AGF30, AGF347	SHMT2	Untested in myeloid malignancies	N/A	(84)
Arginine	CB1158	Arg1, Arg2	Myeloid cells	Blocks myeloid cell mediated inhibition of T-cell and NK cell proliferation	(89)
	BCT-100	Extracellular arginine depletion	Primary AML blast for adult and pediatric patients	Induction of G_0_/G_1_ cell cycle arrest, shortly followed by necrotic cell death	(85)
	ADI-PEG		ASS-1 negative AML patient derived xenograft	Reduced AML burden in tumor bearing mice	(105)
Ornithine	DFMO	ODC	Five AML patients treated in combination with MGBG	Complete response in one patient and partial response in four	(100)
			AML cell line THP1	Induction of cell death characterized by cleavage of PARP, Caspase 3 and Caspase 7	(101)
			CML Cell line K562 or myeloid cells	Decreased Polyamine synthesis and reduction in proliferation	(102)
	AO476	AMD1	Patient derived CML blasts	Activated the integrated stress response and led to a reduction in proliferation	(98)
Asparagine	ASNase	Extracellular asparagine and glutamine depletion	Patient derived AML blasts	Increased toxicity in FAB, M5, M1 and M4 AML subtypes and resistance in M3 and M2 (determined by MTT assays)	(109)
			AML cell line U937; primary AML blasts	Induction of apoptosis *in vitro* and increase survival in U937 xenograft mice	(60, 111)
			Five adult AML patients	Reduction in plasma glutamine and asparagine in all five patients; complete response in two patients, partial response in one	(112)
			Leukemic stem cells	Induction of apoptosis; reduced cytotoxicity when co-cultured with mesenchymal stem cells or macrophages	(113)

## Essential Amino Acids

### Methionine

**Figure 1:**
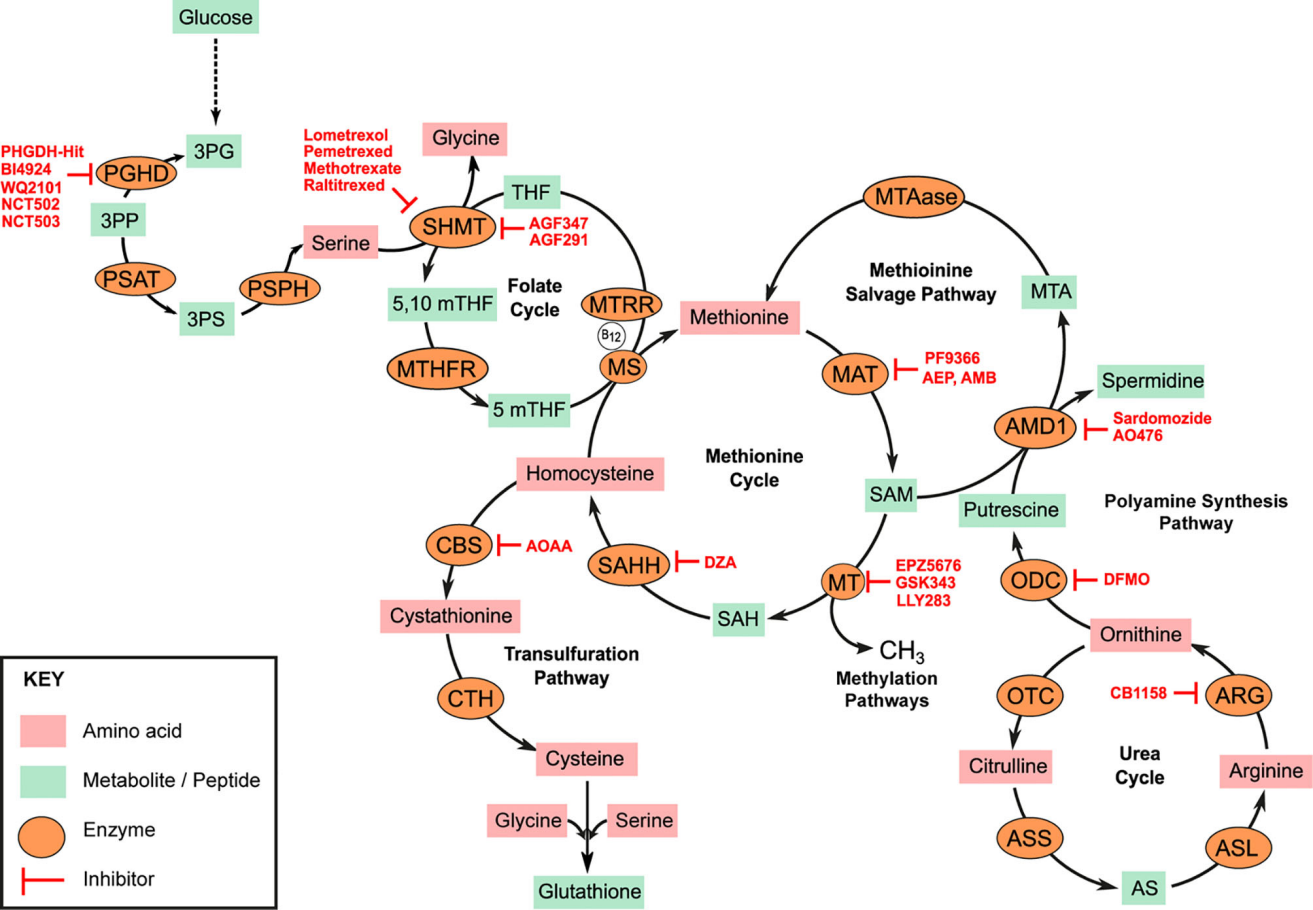
Amino acid metabolic pathways. Serine, Glycine metabolic pathway represents a significant glycolysis deviation pathway in cancer. Overexpression of phosphoglycerate dehydrogenase (PHGDH) drives conversion of 3-phosphoglycerate (3PG) to 3-phosphohydroxypyurvate (3PP). 3PP is aminated to 3-phosphoserine (3PS) by phosphoserine aminotransferase (PSAT). 3PP is subsequently hydrolyzed to serine by phosphoserine phosphatase (PSPH). Serine hydroxy methyltransferase (SHMT) simultaneously catalyzes the conversion of serine to glycine and tetrahydrofolate (THF) to 5,10 methyltetrahydrofolate (5,10mTHF). Methyltetrahydrofolate reductase (MTHFR) catalyzes the reduction of 5,10mTHF to 5mTHF which is critical for the re-methylation of homocysteine to methionine by methionine synthase (MS). MS requires Vitamin B12 as cofactor which must be converted to its co-factorial form by methionine synthase reductase (MTRR). Methionine adenosyl-transferase (MAT) isoenzymes catalyze the conversion of methionine to S-adenosyl methionine (SAM) in an ATP-dependent reaction. SAM is the universal donor of methyl units to several methyl transferases (MTs) in the cell and thus regulates several methylation sensitive reactions such as DNA, RNA, or histone methylation. S-adenosyl homocysteine (SAH) is hydrolyzed to homocysteine for methionine resynthesis by SAH hydrolase (SAHH). Some homocysteine can be deviated to the transulfuration pathway for the generation of cysteine. Homocysteine is first converted to cystathionine by cystathionine beta synthase (CBS) and subsequently to cysteine by cystathionine *γ*-lyase (CTH). Some SAMs are deviated for the synthesis of polyamines. S-adenosyl methionine decarboxylase (AMD1) catalyzes the transfer of aminopropyl groups from SAM to putrescine to generate spermidine and 5-methionineadenosine (MTA). Methionine can be salvaged from this reaction by 5-methionineadenosine phosphorylase (MTAase). ODC, Ornithine decarboxylase; OTC, Ornithine transcarbamylase; ARG, Arginase; ASS, Argininosunicate synthase, AS, Argininosuccinate; ASL, argininosuccinate lyase. Specific enzyme inhibitors (highlighted in red) are discussed in the main text.

Methionine is a neutral non-polar sulfur containing amino acid. It is one of two amino acids required for polyamine synthesis (the other being ornithine). It is a critical component of one-carbon metabolism and a primary source of intracellular methyl units involved in epigenetic modulation of gene transcription and RNA translation. That myeloid leukemias are auxotrophic for methionine has been extensively documented (2–4). Analysis of the plasma and bone marrow of acute myeloid leukemia (AML) patients shows a significant reduction in the abundance of free methionine when compared to healthy controls (3, 4). The elevated overall transmethylation rate in myeloid and other malignancies is widely accepted as the basis for the methionine dependence of cancer cells. Intracellularly, methionine is converted to S-adenosyl-methionine (SAM) in an ATP dependent reaction (**Figure 1**). SAM production from methionine is catalyzed by isoenzymes of the methionine adenosyl transferase (MAT) family also known as S-adenosyl-methionine synthases [**Figure 1**, Markham and Pajares (5)]. These include MAT1, MAT2, and MAT3. MATs are upregulated in AML blasts and the corresponding overproduction of SAM has been correlated to poor prognosis (6). SAM is the universal donor of an active methyl group to several cellular transmethylation dependent epigenetic processes. Histone and DNA hypermethylation is a characteristic feature of myeloid malignancies and several distinct methylation patterns govern the progression and biological behavior of disease (7, 8). SAM is readily demethylated to S-adenosyl-homocysteine (SAH) by several methyltransferases such as the histone methyltransferase DOTL1 (disruptor of telomeric silencing 1-like). Many methyltransferase inhibitors (such as 5-azacytidine, decitabine or 5-fluoro-2-deoxycitidine) currently used in the treatment of MDS and MPN are nucleoside analogs, and thus their permanent incorporation into DNA is often associated with significant long-term toxicities. Alternative strategies now aim to directly disrupt methionine and/or SAM metabolism.

The competitive inhibition of MAT by methionine analogs has been tested. PF-9366 is a potent allosteric inhibitor of human MAT2 shown to inhibit proliferation and decrease the viability of AML cell lines bearing the oncogenic mixed leukemia lineage MLL-AF9 fusion protein (9, 10). 2-amino-4,5-epoxypentanoic acid (AEP), cycloleucine, and 2-Amino-4-methoxy-cis-but-3-enoic acid (AMB) are other small molecules shown to drastically inhibit MAT activity and decrease SAM production in various cancer cell types but are thus far untested against myeloid neoplasms (11–13). Similarly, small molecules known to interfere with SAM interaction with specific methyltransferases have been developed. The amino nucleoside analog Pinometostat (EPZ-5676) competitively inhibits the activity of the DOTL1 by occupying its SAM binding pocket leading to a reduction in H3K9me seen when trailed in both adult and pediatric AML patients (14). Like Pinometostat, GSK-343 is a competitive inhibitor SAM binding to the enzymatic subunit of the histone methyltransferase EZH2 (Enhancer of zeste homolog 2). GSK-343 treatment of the AML cell line Kasumi led to a significant reduction in H3K27me3, G_0_/G_1_ cell cycle arrest and a corresponding reduction in colony size after 96 h treatment (15). Other SAM mimetics such as LLY-283—a PRMT5 (protein arginine methyltransferase) inhibitor—significantly impair AML cell lines MOLM-13 and MV4-11 proliferation *in vitro* (16).

Methionine can be re-synthesized from homocysteine produced from the hydrolysis of SAH by S-adenosyl-homocysteine hydrolases (SAHH, also known as S-adenosyl-homocysteinase; **Figure 1**). Inhibiting methionine re-synthesis provides another avenue for blocking the production of SAM in myeloid leukemias. 3-deaza-adenosine (DZA) is a cyclic dinucleotide based inhibitor of SAHH. DZA treatment of primary AML blasts as well as the myelomonocytic cell line MV411 resulted in elevated intracellular SAH levels and a corresponding decrease in overall methylation potential in these cells (17). Similarly, DZA treatment of the AML cell lines HL60 and U937 induced apoptotic cell death characterized by cleavage of poly ADP-ribose polymerase (PARP) and activation of Caspase 3 (18). The homocysteine to methionine conversion is catalyzed by the enzyme methionine synthase (MS). MS is encoded by the 5-methyltetrahydrofolate-homocysteine methyltransferase (MTR) gene. Polymorphisms in the MTR gene are known to moderate the risk in various leukemias although specific evidence in myeloid neoplasms is limited. However, the downregulation of MS in many methionine dependent cells and the corresponding reduction in *de novo* methionine synthesis in these cells theoretically explain the dependence of methionine import from external sources. MS uses methyl-tetrahydrofolate as a substrate and vitamin B12 as cofactor. MS bound vitamin B12 must be converted to its required methylated co-factorial form by the co-enzyme methionine synthase reductase (MTRR). Polymorphism in the MTRR gene is strongly associated with increase plasma concentrations of homocysteine and an increased risk of AML (19). To explain why children with Down Syndrome (DS) and subsequently diagnosed with AML responded better to the folate and cytosine analogs methotrexate and 1-*β*-D-arabinofuranosyl cytosine (Ara-C) respectively, Ge et al. described a direct correlation between elevated mRNA expression of the enzyme cystathionine-*β*-synthase (CBS) in DS patients and drug sensitivity (20). CBS catalyzes the first step in the transsulfuration pathway required for the conversion of homocysteine first to cystathionine and subsequently to cysteine. Elevated CBS expression in DS-AML patients may lead to a reduction in homocysteine available for the re-synthesis of methionine and subsequently SAM. CBS overexpression has also been reported in the CML cell line K562. However, Wang and colleagues argue that the increased production of sulfides as by-products of CBS activity is tumorigenic; thus the inhibition of CBS activity in K562 and mononuclear cells isolated from the bone marrows of CML patients using the CBS-inhibitor aminooxy acetic acid (AOAA) lead to an overall reduction in proliferation and the induction of apoptosis in these cells (21).

The energy independent cellular import of methionine in methionine dependent cells is predominantly *via* the sodium dependent system B^0,+^ transporters for neutral and basic amino acids (ATB^0,+^/SLC6A14 and B^0^AT1/SLC6A19), the sodium-coupled neutral amino acid transporters (SNAT1/SLC38A1 and SNAT2/SLC38A2), the sodium-optional systems y^+^L transporters for large neutral amino acids (LAT1/SLC7A5, y^+^LAT1/SLC7A7, and y^+^LAT2/SLC7A7). The alanine/serine/cysteine transporter ASCT2/SCL1A5 has also been shown to transport significant amounts of methionine. Given that many solute carriers transport methionine with varying affinities, the complete inhibition of methionine import *via* specific transport blockers is not therapeutically practical. An alternative approach aims to enzymatically degrade extracellular methionine in the blood of MDS, MPN, or AML patients.

Methioninases (L-methionine-*α*-amino-*γ*-mercaptoethane lyase or METase) are pyridoxal-L-phosphate (PLP)-dependent enzymes that cleave methionine and other sulfur containing amino acids to their respective keto acids. Growth inhibition of blasts isolated from AML patients and treated with METase from *Clostridium sporogenes* had been demonstrated almost four decades ago (22, 23). However, development and clinical use of METases have not really progressed despite ample evidence of their preclinical efficacy in reducing plasma methionine levels and stalling cell growth in several methionine dependent cancers. This is possibly because the recombinant methioninases (rMETases) used are cloned from foreign microorganisms such *Clostridium sporogenes, Pseudomona putida* and produced in *Escherichia coli* and thus, are highly immunogenic in man. A PEGylated rMETase (PEG-rMETase) has recently been developed aimed at reducing immunogenicity and improving circulating half-life of the enzyme but has so far not been tested against leukemic cells (24).

### Leucine, Isoleucine, and Valine

**Figure 2:**
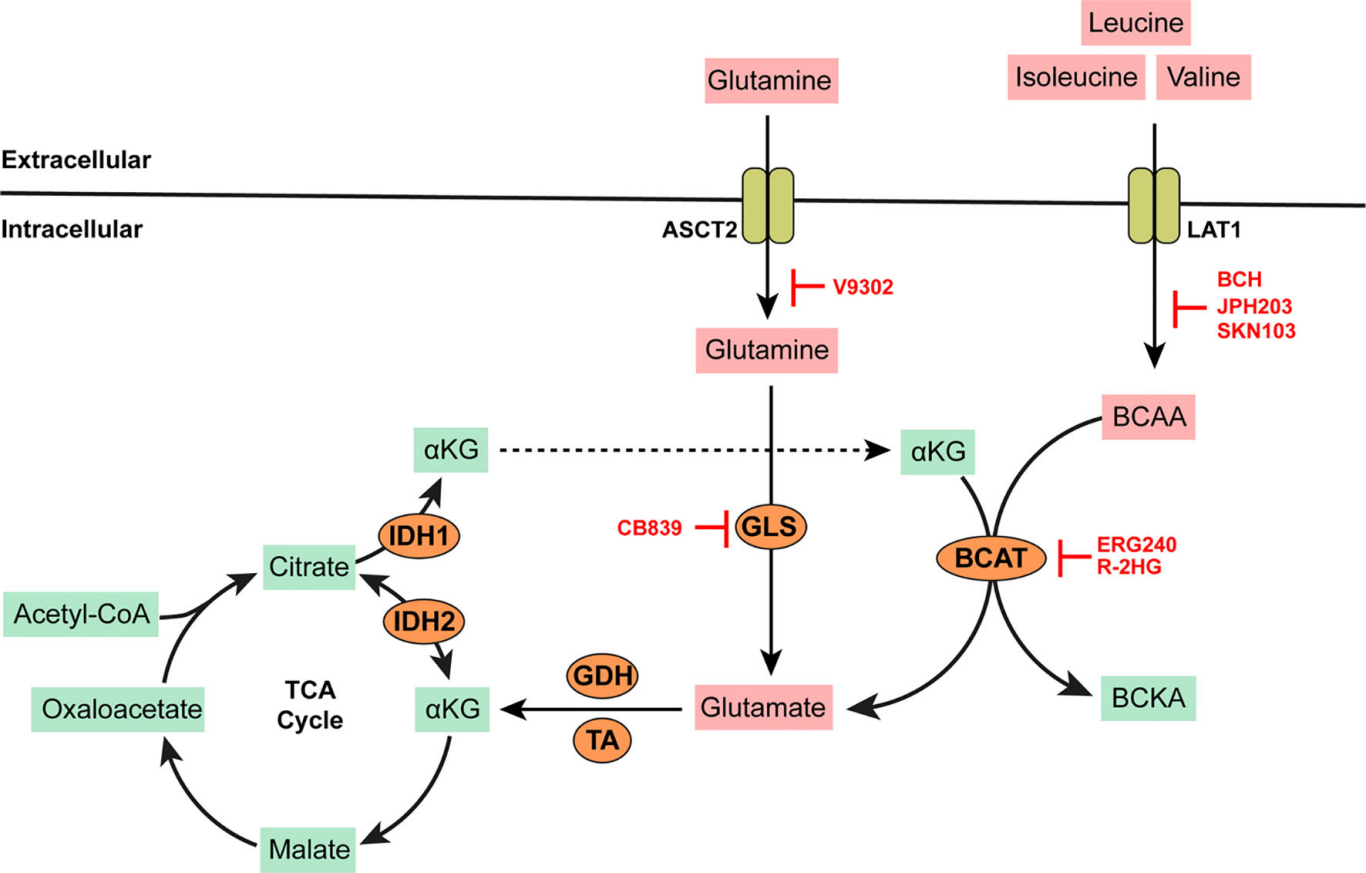
BCAA and Glutamine metabolism. BCAAs such as leucine, isoleucine, and valine are imported into the cell by high affinity transporters such as LAT-1. Branched chain amino acid transaminases (BCAT) catalyze the highly reversible transfer of *α*-amino groups from any BCAA to *α*-ketogluterate (*α*KG) to generate glutamate. More glutamate can be synthesized by the action of mitochondrial glutaminase (GLS) on glutamine imported into the cell *via* the alanine/serine/cysteine transporter (ASCT2, which is capable of high affinity glutamine transport). Glutamate is converted to *α*KG by either glutamate dehydrogenase (GDH) or several amino acid transaminases (TAs) such aspartate aminotransferase (AST) or alanine transaminase (ALT). *α*KG produced in this way is anaplerotic to the tricarboxylic acid (TCA) cycle. *α*KG can be reversibly converted to citrate *via* isocitrate dehydrogenase (IDH) which is overexpressed in some AMLs. AMLs bearing IDH mutations produce excess 2-hydroxygluterate (R-2HG) which can inhibit BCAT activity. All other specific enzyme inhibitors (highlighted in red) are discussed in the main text.

Leucine, isoleucine, and valine are proteinogenic, neutral, branched-chain amino acids (BCAAs) that must be obtained from the diet in humans. They play an essential role in reprogramming energy metabolism and lipogenesis in cancer and promote tumor growth *via* a plethora of pathways. In murine models typifying disease progression across various phases of human CML, the plasma concentrations of BCAAs are significantly lower in mice at the chronic disease phase (CP-CML) when compared to the blast crisis phase (BC-CML) or healthy mice (25). BCAAs are homeostatic regulators of *α*-ketoglutarate (*α*KG) which is produced from isocitrate by isocitrate dehydrogenases (IDH). *α*KG is a pleotropic metabolite with numerous functions, including as an intermediate of the tricarboxylic acid (TCA) cycle and in the regulation of electron transport chain activity (26). One of two branched-chain amino acid transaminases (cytosolic BCAT1 or mitochondrial BCAT2) catalyzes the highly reversible transfer of *α*-amino groups from any of these BCAAs to *α*KG to produce glutamate and their corresponding branched-chain keto acid (BCKA; see **Figure 2**). High levels of BCAT1 mRNA and protein expression have been reported in human AML blasts and leukemic stem cells (LSCs) isolated from bone marrow aspirates of AML patients (27). Similarly, BCAT1 mRNA expression is significantly higher in CML cells (irrespective of disease phase) when compared to normal hematopoietic pluripotent stem cells (HPSC) (25). The elevated expression of BCAT1 has also been implicated in the progression from MPN and MDS to myelofibrosis and leukemic transformation (28, 29). Strategies for the inhibition of BCAT activity has been explored for therapeutic benefit in BCAA dependent leukemias.

The knockdown of BCAT1 in LSCs leads to the accumulation *α*KG, degradation of hypoxia inducible factor (HIF1*α*), and an overall reduction in the growth and leukemia-initiating potential of these cells (27). ERG-240 is a leucine analog that has been identified as a novel potent BCAT1 inhibitor (30). ERG-240 treatment reduces oxygen consumption, glycolysis, and itaconate levels in myeloid cells (30, 31). 2-hydroygluterate (R-2HG) is a naturally occurring enantiomer with structural similarity to *α*KG and thus inhibits the unidirectional *α*KG-dependent BCAT1 activity (32). However, inhibition *via* R-2HG leads to compensatory glutamate catabolism and the activation of alternative transaminases. It is also important to note that R-2HG is naturally produced by several cancers bearing IDH mutations. Heterozygous IDH mutations are predicted to occur in up to 20% of AML patients. Thus, additional BCAT inhibition *via* R-2HG may provide no additional anti-cancer benefit in these patients. Indeed, low concentrations of BCKAs have been described in IDH-mutated AML blasts with a corresponding increase in intracellular BCAAs such as leucine. BCAA production in these cells is dependent on glutamate availability and mediated by the reverse activity of BCAT1 not inhibited by R-2HG or any available BCAT inhibitors.

In BCAA dependent tumor cells, BCAAs are imported into the cell *via* system L transporters (LAT1/SLC7A5, LAT2/SLC7A8, LAT3/SLC43A1) and the system b^(0,+)^ transporter b^(0,+)^AT1/SLC7A9. Leucine transport *via* these transporters is critical for the constitutive activation of the nutrient sensing phosphatidynol-3-kinase (PI3K) and mammalian target of rapamycin (mTOR) pathways in myelogenous leukemias (33). Gene expression analysis of mononuclear cells isolated from the bone marrows of a cohort of MDS patients (at various stages of disease progression) reveals that the upregulation of SLC7A5 gene transcription and LAT1 protein is a characteristic feature of leukemic evolution (34, 35). Similarly, CRISPR-based screens of 14 human AML cell lines identified SLC7A5 as an essential gene for cell survival (36). siRNA mediated silencing of SLC7A5 in the MDS cell line SKM-1 leads to cell cycle arrest, reduced proliferation and the induction of apoptosis in these cells (35). System L transporters LAT1, LAT2, and LAT3 mediate the reversible import of leucine in exchange for glutamine across the cell membrane and have emerged as useful anti-cancer targets due to their central role in both leucine and glutamine metabolism. Several small molecules that inhibit system L transport activity and blunt tumor cell growth have been developed.

The anti-leukemic properties of leucine analogs such as 2-aminobicyclo-(2,2,1)-heptane-2-carboxylic acid (BCH) have been documented (37, 38). BCH inhibits leucine import *via* LAT1, LAT2, and LAT3 and significantly slows the *in vitro* growth of the AML cell lines HL60 and NB4, as well as the CML cell line K562 (39). BCH treatment leads to a reduction in phosphorylation of the S6 ribosomal protein (S6RP) involved in the activation of the mTOR pathway in these cells (40). 2-amino-3(4[methoxy]-3,5-dichlorophenyl) propanoic acid (JPH203, previously known as KYT-0353) is another potent selective inhibitor of leucine transport *via* LAT1 (38). Ongoing work from our lab is investigating the effect of JPH203 on AML pathogenesis. (S)-2-amino-3-(4-((7-(3-aminophenyl)naphthalen-1-yl)methoxy)-3,5-dichlorophenyl)propanoic acid (SKN103) is another non-transportable blocker of LAT1 currently being developed (41). SKN103 blocks leucine transport and mTOR activation in several cancer cell lines but remains untested against myeloid malignancies. Using computer modeling techniques, Massaro and colleagues identified several new small molecules capable of a dose dependent reduction of both leucine and methionine import *via* LAT1 (42). However, further work is needed to ascertain the anti-cancer efficacies of these molecules. Recently, Udea and colleagues described the generation of a novel anti-human LAT1 monoclonal antibody based on the rat Ig2a heavy chain which when bound to human LAT1 blocked the uptake of BCAAs and blunted tumor growth (43).

### Tryptophan

**Figure 3:**
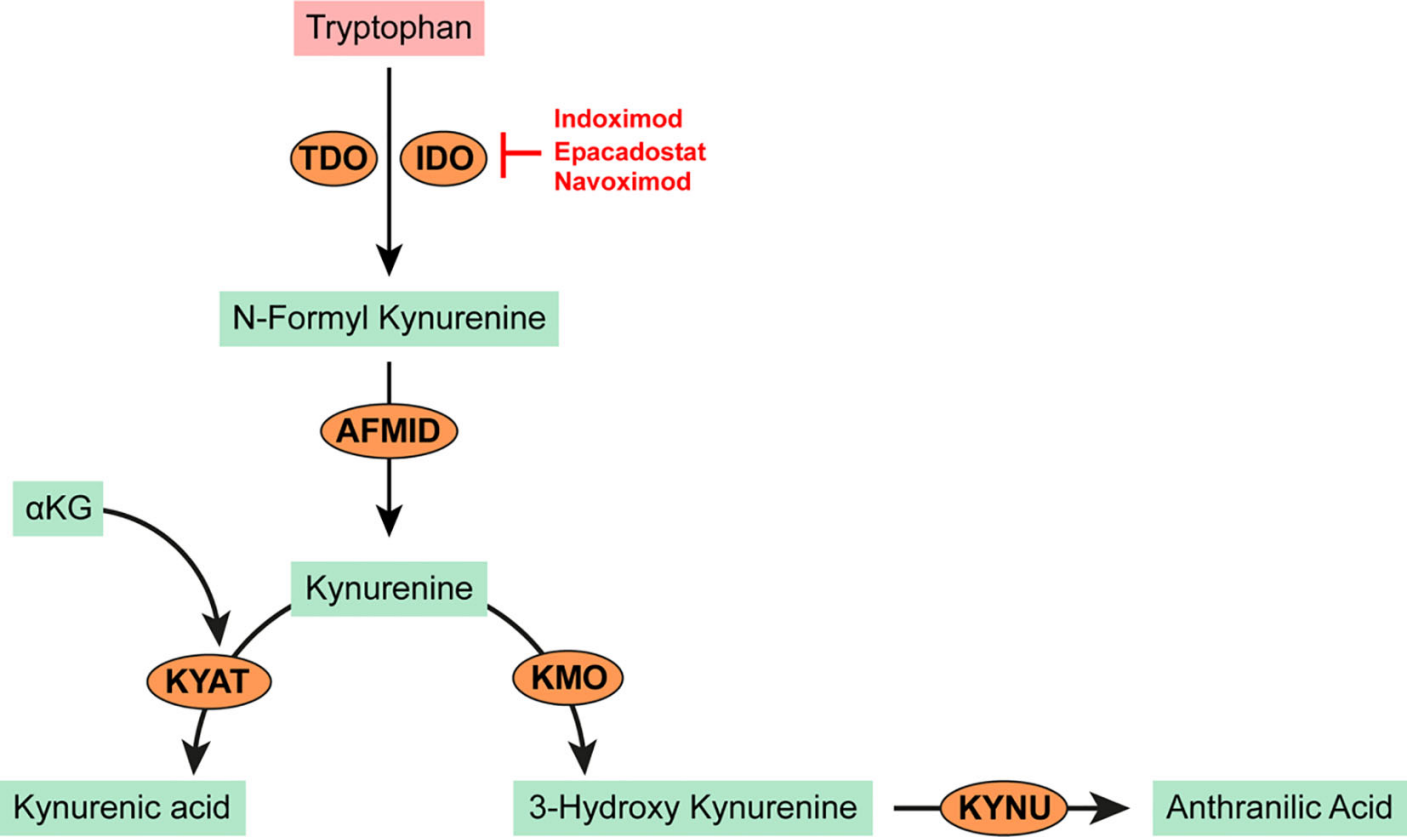
Tryptophan metabolism in cancer. Tryptophan is oxidized by the heme-dependent endocellular enzymes tyrptophan 2,3 dioxygenase (TDO) or indoleamine 2,3 dioxygenase (IDO) to produce N-formyl kynurenine. N-formyl kynurenine is hydrolyzed by kynurenine formidase (AFMID) to kynurenine. Kynurenine may be further transaminated to kynurenic acid by kynurenine-oxoglutrate transaminase (KYAT) or oxidized to 3-hydroxy kynurenine by kynurenine 3-monooxygenase (KMO). Kynureninase (KYNU) catalyzes the cleavage of either kynurenine or 3-hydroxy kynurenine to anthranilic acid which is toxic to T and NK cells and promotes the recruitment of MDSCs.

The tryptophan metabolic pathway has garnered significant research interest in the search for therapeutic targets due to its prominent role in cancer immunity. The heme-dependent endocellular enzymes tryptophan 2,3 dioxygenase (TDO) or indoleamine 2,3 dioxygenase (IDO) catalyze the oxidation of tryptophan to produce N-formylkynurenine which is subsequently hydrolyzed to kynurenine. IDO mediated depletion of tryptophan and accumulation of kynurenine or its downstream derivatives (*e.g.* kynurenic acid, 3-hydroxy-kynurenine, anthranilic acid; see **Figure 3**) are toxic to both T and NK cells. Kynurenine also promotes a phenotypic switch of immune cells to immunosuppressive phenotypes; for example, from T cells to Tregs or from M0 macrophages to M2 phenotypes (44–46). Tryptophan and kynurenine are critical to the recruitment and expansion of myeloid derived suppressor cells (MDSCs) in many cancers. Consequently, high levels of serum kynurenine predict poor outcome in AML patients (47). In IDH mutated AMLs, kynurenine-3-monoxygenase (KMO) and kynureninase (KNU) enzymes (which are required for the catalytic cleavage of kynurenine to L-alanine and anthranilic acid) are significantly downregulated leading to further accumulation of kynurenine (48). IDO is constitutively overexpressed and secreted from primary AML blasts (49–51). Its activity has been linked to poor prognosis in patients with MDS and secondary leukemia (51–55). Plasmacytoid dendritic cells from the bone marrow biopsies of CML patients express high levels of IDO with a strong correlation to disease progression (56). Research aimed at modulating or disrupting the tryptophan/kynurenine pathway has led to the development and use of several specific IDO inhibitors.

Indoximod (1-methyl-D-tryptophan or D1MT) is an orally administered IDO inhibitor (57). Pre-treating AML blasts with indoximod reversed their suppressive effect on T cell proliferation in *in vitro* co-culture experiments (44). Indoximod is currently being tested as a combination therapy with Idarubicin and Cytarabine for newly diagnosed patients with AML in a phase I trial (NCT02835729). Epacadostat (INCB024360) is another orally bioavailable IDO inhibitor tested in patients with advanced MDS after azacytidine treatment (58). In this study, epacadostat treatment resulted in stable disease in about 80% of patients and a marginal reduction in the prevalence of MDSCs and regulatory T-cells. Navoximod (GDC0919, previously NLG919) is an IDO inhibitor shown to restore T-cell proliferation when added to co-culture experiments with IDO expressing human monocyte-derived dendritic cells (59). Lirondostat (BMS986205) is an orally available IDO inhibitor currently being tested in combination with Nivolumab in a phase II clinical trial (NCT02935634) for patients with a diagnosis or history of MDS or AML.

## Conditionally Essential Amino Acids

### Glutamine and Cysteine Metabolism

Glutamine is the most abundant amino acid in the blood. Although it can be synthesized by most mammalian cell types, increased glutamine supply from external sources is required to support rapidly growing cancer cells. In cancer cells, glutamine can be used as a source for carbon and nitrogen, as well as a precursor for the *de novo* biosynthesis of glucose and other amino acids. Willems et al. showed that in addition to inducing apoptosis in AML blasts, glutamine removal from the culture media inhibited leucine uptake and leucine dependent activation of the mTOR pathway (60). Glutamine is converted to glutamate by several enzymes containing glutamine amido-transferase (GATase) domains. Glutamate can also be produced from glutamine *via* mitochondrial glutaminases (GLS1 or GLS2). GLSs are significantly overexpressed in AML, and their allosteric inhibition using the bis-2-(5-phenylacetamido-1,2,4-thiadiazol-2-yl)ethyl sulfide (BPTES) derivative CB-839 (now commercialized as Telaglenastat by Calithera Biosciences) significantly reduces intracellular glutamate titers with a corresponding reduction in cell viability (61, 62). New data points to a decrease in mitochondrial respiration and induction of apoptosis in AML cell lines after treatment with CB-839 (63–65). CB-839 has also been tested in combination with 5-azacytidine in a phase I/II clinical study for patients with advanced MDS resulting in a 70% complete response (66). However, preliminary results of a phase I trial of relapsed AML patients using CB-839 as monotherapy (NCT02081927) showed no objective response (67). Sensitivity of AML cells to CB-893 is increased when administered in conjunction with the FLT3 inhibitor AC220 which is known to alter metabolism in AML cells bearing the oncogenic FMS-like tyrosine kinase 3 (FLT3) and IDH mutations away from glucose towards glutamine catabolism (68).

Glutamine and or glutamate is imported into cells *via* several transporters including system L and y^+^ transporters (LAT1, y^+^LAT1, and y^+^LAT2), the cysteine/glutamate exchanger xCT/SLC7A11, but most significantly, *via* the antiport activity of the sodium dependent neutral amino acid transporter ASCT2/SLC1A5. ASCT2 plays a critical role in promoting leukemia progression. Lineage negative bone marrow cells isolated from the bone marrows of ASCT2^−/−^ heterozygous knockout mice and transduced to include the AML oncogene MLL-AF9 failed to proliferate in xenograft mice models (69). In this study, inhibition of glutamine flux in AML cells using the competitive ASCT2 inhibitor 2-amino-4-bis(aryloxy benzyl)amino butanoic acid (V-9302) led to a reduction in glutamine and leucine uptake and the inhibition of mTOR, ultimately resulting in cell death. ASCT2 can also mediate the high affinity influx of cysteine.

Glutamate transport is intricately linked to intracellular cysteine levels due to the transport stoichiometries of several glutamate/cysteine exchangers. Metabolic profiling of the plasma of AML-M2 patients showed significant reduction in the bioavailability of cystine—a reduced form of cysteine—when compared to healthy controls (3). The elevated consumption of cystine by AML cells is attributed to the overexpression of the system $X_{c}^{-}$ cystine/glutamate antiporter xCT/SLC7A11 in these cells (70). Intracellular availability of the semi-essential amino acid cysteine is rate-limiting to the production of the tripeptide glutathione (GSH). GSH is a major cellular antioxidant that is indispensable to many cancer cells. Given the high levels of ROS production in cancer cells, the import of cysteine for the synthesis of GSH in LSCs is imperative (71). Inhibition of cystine uptake in AML cell lines using the potent xCT inhibitor Erastin leads to the accumulation of intracellular reactive oxygen species (ROS), ultimately leading to cell death by ferroptosis (72, 73). However, several negative regulatory pathways of ferroptosis after Erastin treatment of myeloid cells have been reported, suggesting xCT inhibition alone using single agents may not be sufficient in these settings.

### Serine and Glycine and One Carbon Metabolism

In addition to cysteine, serine and glycine are the principal constituents of GSH required for redox homeostasis in cancer cells. Removal of serine alone from the culture media resulted in a significant decrease in the growth of HL60 leukemia cells (74). Intracellular *de novo* serine and glycine biosynthesis represents a major glycolysis deviating pathway in many cancers (illustrated in **Figure 1**). Stable isotopic tracing revealed increased levels of [2-^13^C] glycine and [2-^13^C] serine in the leukemic cell lines MOML13 and K562 when cultured in media containing [2-^13^C] fructose and [2-^13^C] glucose (75). Elevated serine and glycine intracellular titers in these cells were attributed to increased activity of 3-phosphoglycerate dehydrogenase (PHGDH). PHGDH is the first enzyme of the serine biosynthetic pathway that catalyzes the nicotinamide adenine dinucleotide (NAD^+^)-dependent conversion of 3-phosphoglycerate (3-PG) to 3-phosphohydroxypyruvate (3-PP). The subsequent transamination of 3PP by phosphoserine aminotransferase (PSAT) generates phosphoserine (3-PS) and *α*KG. Phosphoserine is hydrolyzed by phosphoserine phosphatase (PSPH) to produce Serine. ATP-P2x7 signaling in LSCs isolated from the bone marrow of AML patients has been shown to activate PHGDH activity in a cAMP response element binding protein (CREB) dependent manner (76). Blocking PHGDH activity in leukemic cells grown in fructose and glucose containing conditions markedly reduced their cell growth (75). AML bearing internal tandem duplications of FLT3 also have elevated expression of PHGDH (77). The transamination of 3-PP by PSAT is anaplerotic to the TCA and requires glutamine as the amino acid donor. Serine metabolism is therefore intricately coupled to glutamine metabolism. Inhibition of glutamine metabolism in AML leads to the upregulation of both PHGDH and PSAT in HL60, K562, and THP1 cell lines (74). The removal of both serine and glutamine from culture conditions significantly blunts AML cell growth *in vitro*. Inhibition of *de novo* serine synthesis *via* the PHGDH inhibitor WQ-2101 leads to induction of apoptosis in MV411, MOLM13, and PL21 cell lines, all of which harbor FLT3-ITD mutations but not OCI-AML3, Kasumi-1, or HL60 (77). Other PHGDH inhibitors shown to reduce proliferation of serine addicted cancer (but remain as of yet untested in AML, MDS, or MPN) include the pro-drug BI-4924, PHGDH-Hit, NCT502, and NCT503 (78, 79).

Serine hydroxyl methyl transferase (cytosolic SHMT1 or mitochondrial SHMT2) catalyzes the reversible conversion of serine to glycine. Higher expression of SHMT1 has been shown in mononuclear cells isolated from the blood of AML patients when compared to age-matched healthy individuals (80). Similarly, expression of SHMT1 and SHMT2 is increased in serine-dependent AML cell lines most of which bear the MLL-AF9 fusion protein (81). The reversible conversion of serine to glycine by SHMTs is dependent on tetrahydrofolate. Using the human CML cell line HAP1, Tranmonti et al. reported a significant reduction in the live cell counts of HAP1 cells transduced with shRNAs against SHMT2 (82). Additionally, treatment of these cells with Compound 12.2—a pyrazolopyran derivative—led to further reduction in the viabilities of both wild-type and SHMT2-knockout cells. Scaletti et al. have recently described evidence for the indirect inhibition of human SHMT activity by the clinically available folate analogs Pemetrexed, Lometrexol, Methotrexate, and Raltitrexed (83). Among these, Lometrexol was the most active, reducing recombinant human SHMT1 activity by over to 60% and SHMT2 by 50%. Although these drugs already have demonstrable antileukemic efficacy, direct evidence of the inhibition of SHMTs in myeloid leukemia is lacking. Other potent SHMT2 inhibitors currently being developed include the pyrrolo[3,2-d] pyrimidine compounds AGF291, AGF30, and AGF347 all of which remain untested in myelogenous leukemias (84).

Serine and glycine are major donors of one-carbon units in mammalian cells. The oxidative decarboxylation of glycine by mitochondrial enzymes of the glycine cleavage system (GCS) is an important source of one carbon units for several downstream processes. GCS comprises of four proteins. These are T-protein (GCS-T), P-protein (also known as glycine dehydrogenase GLDC), L-protein (GCS-L), and H-protein (GCS-H). The methylamine produced following glycine decarboxylation by GLDC is transferred to amino-methyl transferase (GCS-T) which catalyzes the release and transfer of a methyl group to tetrahydrofolate producing 5,10-methylene tetrahydrofolate (mTHF). mTHF produced from this reaction is critical to the synthesis of methionine from homocysteine and regeneration of SAM. mTHF produced from the GCS can be further reversibly oxidized by methylene tetrahydrofolate dehydrogenase enzymes (cytosolic MTHFD1 or mitochondrial MTHFD2) to 10-formyl tetrahydrofolate which is important for nucleotide synthesis. shRNA mediated knockout of MTHFD2 in three human AML cell lines and mouse MLL-AF9 leukemia cells leads to a significant decrease in cell growth and impaired leukemogenesis *in vivo* (81).

### Arginine, Ornithine and Polyamine Synthesis

In addition to its role in protein synthesis, nitric oxide production, and creatinine synthesis, arginine is an important source of ornithine for polyamine synthesis. Positively charged naturally occurring polyamines such as spermidine and spermine interact with various negatively charged macromolecules (DNA, RNA, proteins) in the cell. Polyamine availability therefore regulates several processes that promote cell growth, proliferation, differentiation, migration, and survival. For polyamine synthesis, arginine is first hydrolyzed to ornithine and urea by one of two isoenzymes (cytosolic Arg1 and mitochondrial Arg2). Arg2 expression and activity are elevated in AML blasts leading to a rapid reduction in plasma arginine concentrations (85, 86). Similarly, Arg1 is overexpressed and highly active in monocular cells isolated from the bone marrow aspirates of patients with lower-grade/lower risk MDS and CML (87). Transgenic B6D2F1 mice constitutively expressing Arg1 downstream of a CD68 promoter showed increase incidence of myeloid leukemia and myeloid dysplasia when compared to wild-type controls (88). Potent arginase inhibitors such as CB-1158 (Calithera Biosciences) have been developed and proposed as monotherapies in cancers with elevated arginase expression, but however remains untested against myeloid cancers. A consequence of elevated arginase expression and depletion of extracellular arginine is the immunosuppression of T cells. CB-1158 has recently been shown to block myeloid cell mediated inhibition of T-cell and NK cell proliferation (89).

Ornithine produced by arginase activity is subsequently decarboxylated by ornithine decarboxylase (ODC) to produce the polyamine precursor putrescine. Aminopropyl groups are then transferred to the primary amine groups of putrescines by S-adenosyl methionine decarboxylase (AMD1 or SAMDC) to generate spermidine. Spermine is produced from spermidine and decarboxylated S-adenosylmethionine (dcSAM) by spermine synthase (SMS). AMD1 upregulation has recently been implicated in CML progression from chronic to blast crisis phase and associated with poor prognosis in AML (90). The deletion of AMD in patient derived CML blasts or inhibition of AMD1 in K562 cells inhibited their proliferation *in vitro* as well as impaired engraftment *in vivo*. Similarly, ODC activity was significantly higher in whole leukocyte extracts from patients with CML compared to healthy controls (91). Overexpression of AMD1, ODC1, and SMS in AML is regulated by the extended signaling network of the MYC oncoprotein, which is amplified in over 90% of AML patients (92–94). Indeed, disruption of MYC signaling in the human AML cell lines MOLM-13, HL60, THP1, and KG1a leads to a marked downregulation in AMD1expression (95). Phosphorylation of AMD1 at Ser298 by activated mTORC1 further stabilizes the enzyme and increases its half-life in the cell (96). AMD1 has thus emerged as a central target for inhibiting polyamine synthesis in cancer.

Sardomozide (previously denoted CGP48664A or SAM486A) is a methylglyoxal-bis guanyl hydraxone (MGBG) derivative that selectively binds to and inhibits AMD1 activity. Early phase 1 trials investigating the clinical tolerability of Sardomozide in patients with various advanced solid cancers showed significant myelosuppression was the main dose limiting toxicity among many others (97). These toxicities have since stymied interest in progressing Sardomozide for clinical use against myeloid leukemias in favor of other novel AMD1 inhibitors. Using computer assisted drug design techniques, Liao et al. recently identified a novel AMD1 inhibitor (denoted AO476/40672079) which showed significant inhibitory potency against recombinant human AMD1 (98). Treating patient derived CML blasts with AO476 activated the cell stress response and led to a reduction in cell proliferation, as well as suppressed the growth of nilotinib-resistant CML *in vivo* without any significant toxicities (90). The naturally occurring ODC antienzymes (OAZ1, OAZ2, and OAZ3) negatively regulate ODC activity in response to elevated intracellular polyamine levels. However, analysis of the mRNA levels of OAZ1 in some leukemias points to a significant downregulation in its expression (99). Alternative strategies for inhibiting ODC activity in cancer cells have been explored. D,L-alpha-difluoromethylornithine (DFMO or eflornithine) synthesized over 30 years ago is a specific irreversible inhibitor of ODC (and to a lesser extent Arg1 and Arg2 by feedback inhibition by the accumulation of ornithine). The treatment of five AML and five CML patients with DFMO in combination with MGBG led to complete response in one patient and partial response in four (100). Treating the AML cell line THP1 with DFMO led to PARP, caspase 3, and caspase 7 cleavage indicative of the induction of cell death (101), Similarly DFMO treatment decreased proliferation of the CML cell line K562 (102). While the preclinical efficacy of DFMO to reduce intracellular polyamine levels and impair proliferation of myeloid cells (103) and leukemic cells has effectively been shown, there is no clinical data on its efficacy in myeloid malignancies.

An alternative strategy for the inhibition of polyamine synthesis in malignant cells is to directly impair the supply of arginine needed to produce ornithine. Arginine is non-essential to non-malignant cells due to their intrinsic ability for arginine re-synthesis from other amino acids. The main pathway for arginine re-synthesis is *via* enzymes of the urea cycle, including argininosuccinate synthase (ASS) that catalyzes the conversion of citrulline to argininosuccinate, and argininosuccinate lyase (ASL) required for the conversion of argininosuccinate back to arginine (104). In malignant cells a significant downregulation of enzymes of the arginine re-synthesis pathway exacerbates their dependency on extracellular arginine, which is imported into the cell *via* the high affinity cationic amino acid transporters (CAT1/SLC7A1). Work by Miraki-Moud and colleagues reveals extensive methylation of the ASS1 promoter and downregulation of ASS1 expression in about 75% of AML samples studied (105); ASS1 downregulation was correlated to elevated CAT1 expression in these samples. Consequently, AML blasts cultured in arginine depleted conditions *in vitro* leads to the induction of G_0_/G_1_ cell cycle arrest shortly followed by necrotic cell death (85).

Several extracellular arginine degrading enzymes have been developed. Arginine deiminase (ADI) is a *Mycoplasma sp* derived arginine depleting enzyme developed for therapeutic use. Therapeutic ADI is PEGylated to reduce its immunogenicity and improve circulatory half-life. ASS-1 negative AML blasts were particularly sensitive to ADI-PEG treatment when tested *in vitro*, and when administered in combination with cytarabine, they reduced AML burden in murine models of disease (105). Similarly, BCT-100 is another therapeutic PEGylated human recombinant Arg1 with sequence modifications to improve its catabolic capacity for arginine and increase circulatory half-life. BCT-100 significantly reduced the viability of AML blasts *in vitro* and correspondingly reduced *in vivo* blast count in murine models of disease (85). Other arginine degrading enzymes such as *Escherichia coli* derived arginine decarboxylase (rADC) are available, but are thus far untested in human cancers (106).

## Non-Essential Amino Acids

### Asparagine

Intracellular asparagine is synthesized by the ATP-dependent transamidation of aspartic acid by the cytoplasmic enzyme asparagine synthase (ASNS). In some AMLs, aberrant arrangements at the distal arm of chromosome 7 lead to downregulation of the ASNS gene located at 7q21.3 (107). The downregulation of ASNS and resulting reduction in intracellular asparagine synthesis in these AML blasts render them particularly sensitive to asparagine depletion. Asparagine, when incorporated into peptide sequences, provides critical sites for various post translational modifications that determine protein structure. As such, short term removal of asparagine activates the unfolded protein response (UPR) that can lead to cell death if asparagine levels are not restored (108). Intracellular asparagine is also an important exchange factor required for the antiport of several amino acids such as glutamine, serine, histidine, and arginine, and as such disrupting asparagine metabolism can result in a significant reduction in the transport and intracellular levels of these conditionally essential amino acids.

Bacterial derived L-asparaginase (ASNase) is a well-established active asparagine-depleting agent used for the treatment of several childhood leukemias and lymphomas, although data on myeloid leukemias is scant. In a large study, Okada et al. found that the FAB, M5, M1, and M4 subtypes of AML were particularly sensitive to ASNase treatment while the M3 and M2 subtypes were resistant (109). Sensitivity to ASNase is ultimately linked to intrinsic asparagine synthetase activity, with M5 AML subtypes known to show the lowest (110). ASNase treatment of the AML cell line U937 led to the induction of apoptosis in these cells and improved the survival of mice inoculated with U937 cells (111). ASNases also have glutamine catabolic activity depending on their origin. *Erwinia chrysanthemi* derived ASNase (periplasmic, type II ASNase) contains significantly higher glutaminase activity than cytoplasmic type I variants isolated from *Eschericial coli*. Glutamine depletion by *Erwinia* ASNase leads to inhibition of mTORC1 and induction apoptosis in primary AML blasts (60). Similarly, treatment of five adult AML patients with *Erwinia* derived ASNase led to significant reduction of plasma glutamine and asparagine and resulted in complete response in two patients and a partial response in one (112). Michelozzi and colleagues recently showed that AML blasts and LSC were particularly sensitive to *Eschericia coli* or *Erwinia* derived ASNase treatment (113); however cytotoxity of ASNase was greatly reduced when LSCs were co-cultured with bone marrow derived mesenchymal stem cells or macrophages which can resynthesize and release asparagine into the bone marrow niche.

## Conclusions

Amino acid availability in the immediate microenvironment of myeloid cancer cells strongly modulates cancer cell growth and viability. In this review we discussed advances in our understanding of dysregulated amino acid metabolism in cancer cells and the significant developments in therapeutics capable of disrupting amino acid metabolic pathways that are critical to cell growth. Specific transport inhibitors were some of the early therapeutics to emerge. However, the redundancy in transport systems (*i.e.* multiple high affinity transporters for the same amino acid) reduces the efficacy of these agents as monotherapies. This is reflected in the very few classes of amino acid transport blockers approved for clinical use. In recent decades, new evidence on the genetic and epigenetic background of myeloid malignancies has highlighted a host of transporters and metabolic enzymes that are either overexpressed or silenced and contribute to disease pathogenesis. For instance, the overexpression of MATs in AMLs bearing the oncogenic fusion protein MLL-AF9 or that AMLs with IDH mutations is auxotrophic for glutamine. Such new insights have paved the way for use of specific therapeutic agents capable of blocking the activity of overexpressed enzymes or alternatively use recombinant enzymes to degrade extracellular amino acids. The *in vitro* efficacy of these drugs in abrogating cancer cell growth by shunting specific metabolic pathways has been well demonstrated and preliminary data from phaseI/II clinical trials in man using enzyme inhibitors as single agents or in combination with existing chemotherapies is developing.

AA metabolism by cancers also has a profound effect on the differentiation and functioning of the anti-cancer immune response. We and others have shown that the significant consumption of arginine by AML blasts significantly blunts the proliferation and function of T cells and CAR-T cells. This is also true for other AAs such as tryptophan, methionine, and BCAAs. Based on these observations, a new class of therapeutics aimed at boosting the functioning of the immune system in low amino acid milieus is being developed. For instance, we recently demonstrated the increase *in vivo* persistence and efficacy of anti-CD33 CAR-T metabolically enhanced by inserting ASS1 and OTC expression domains such that these cells could re-synthesized arginine from citrulline discarded by the tumor cells (114). Other agents capable of degrading or depleting the accumulation of tryptophan metabolites such as kynurenine and anthranilic acid (such as KYAT inhibitors) show great promise in blocking the recruitment of regulatory T cells or in suppressing MDSCs (115).

Our improved understanding of the dynamic metabolic relationship between subsets of cells in the tumor milieu highlights a few challenges. Evidence suggests that therapeutics such as ASNase are less effective in inducing cell death in glutamine addicted leukemic stem cells when cultured in the presence of bone marrow derived mesenchymal stem cells or macrophages capable of producing and releasing excess glutamine and asparagine. It is also well documented that fibroblast cells in the bone marrow protect leukemia cells from chemo and immunotherapies. Given that no tumor cell exists in isolation, there is an imperative need to decouple the relationships between amino acid metabolism in tumor cells and other cells found in the bone marrow to better maximize the efficacy of amino acid disruption strategies.

## Author Contributions

LF and LG wrote the manuscript. CS and FM supervised and edited the works and secured funding. All authors contributed to the article and approved the submitted version.

## Funding

We thank R and S Martin and Carter the Brave, in conjunction with Birmingham Children’s Hospital Research Fund, for their funding to support this work.

## Conflict of Interest

The authors declare that the research was conducted in the absence of any commercial or financial relationships that could be construed as a potential conflict of interest.
